# ASSESSING INTERPROFESSIONAL ORAL HEALTH EDUCATION AND PRACTICE FACILITATION OUTCOMES WITHIN LONG-TERM CARE

**DOI:** 10.1093/geroni/igac059.2033

**Published:** 2022-12-20

**Authors:** Jennifer Crittenden, Rachel Coleman, David Wihry, Labrini Nelligan, Denise O'Connell

**Affiliations:** UMaine Center on Aging, Bangor, Maine, United States; University of Maine, Bangor, Maine, United States; University of Maine, Orono, Maine, United States; Lunder-Dineen Health Education Alliance of Maine, Bangor, Maine, United States; Lunder-Dineen Health Education Alliance of Maine, Bangor, Maine, United States

## Abstract

Older adults residing in long-term care (LTC) settings are at an increased risk of poor oral health due to comorbidities and limited ability to provide self-care. Routine oral care is essential for maintaining overall health. Coupled with resident vulnerability is a traditional lack of training and focus on oral health care in LTC. MOTIVATE (Maine’s Oral Team-Based Initiative: Vital Access to Education) is a pilot (N = 8 sites) interprofessional education program focused on daily oral health care within LTC, providing education and technical assistance to advance staff knowledge, skills, and attitudes about oral health. An evaluation was carried out using a pre/post survey design with instruments administered immediately before and after learning module completion along with a survey administered one month after implementation. Knowledge, attitudes, and oral health practices were assessed. A statistical comparison between baseline (N = 491) and post-launch (N = 215) scores revealed a statistically significant improvement (p < 0.001) across all knowledge and attitudes measures including the perceived importance of oral health, understanding of interprofessional roles among the care team, the role of oral health in supporting resident dignity and quality of life, and confidence in providing oral health care. Factors facilitating the transfer of knowledge to practice (N = 478) included personal interest in the topic (46.4%), knowing where to obtain information when needed (47.6%), and knowing how to apply learning to LTC daily care responsibilities (62.4%). Findings underscore the importance of oral health training and implications for practice transformation in an interprofessional context.

